# Secretin modulates appetite via brown adipose tissue-brain axis

**DOI:** 10.1007/s00259-023-06124-4

**Published:** 2023-02-11

**Authors:** Lihua Sun, Sanna Laurila, Minna Lahesmaa, Eleni Rebelos, Kirsi A. Virtanen, Katharina Schnabl, Martin Klingenspor, Lauri Nummenmaa, Pirjo Nuutila

**Affiliations:** 1 Department of Nuclear Medicine, Pudong Hospital, Fudan University, Shanghai, China; 2 Department of Nuclear Medicine, Huashan Hospital, Fudan University, Shanghai, China; 3 Turku PET Centre, University of Turku, Turku, Finland; 4 Turku PET Centre, Turku University Hospital, Turku, Finland; 5 Heart Center, Turku University Hospital, Turku, Finland; 6 Department of Medicine, University of Turku, Turku, Finland; 7 Department of Endocrinology, Turku University Hospital, Turku, Finland; 8 Chair for Molecular Nutritional Medicine, Technical University of Munich, TUM School of Life Sciences, Freising, Germany; 9 EKFZ—Else Kröner Fresenius Center for Nutritional Medicine, Technical University of Munich, Freising, Germany; 10 ZIEL—Institute for Food & Health, Technical University of Munich, Freising, Germany; 11 Department of Psychology, University of Turku, Turku, Finland

**Keywords:** Secretin, Satiation, Neurometabolic coupling, Inhibition, PET, fMRI

## Abstract

**Purpose:**

Secretin activates brown adipose tissue (BAT) and induces satiation in both mice and humans. However, the exact brain mechanism of this satiety inducing, secretin-mediated gut-BAT-brain axis is largely unknown.

**Methods and results:**

In this placebo-controlled, single-blinded neuroimaging study, firstly using [^18^F]-fluorodeoxyglucose (FDG) PET measures (*n* = 15), we established that secretin modulated brain glucose consumption through the BAT-brain axis. Predominantly, we found that BAT and caudate glucose uptake levels were negatively correlated (*r* = -0.54, *p* = 0.037) during secretin but not placebo condition. Then, using functional magnetic resonance imaging (fMRI; *n* = 14), we found that secretin improved inhibitory control and downregulated the brain response to appetizing food images. Finally, in a PET-fMRI fusion analysis (*n* = 10), we disclosed the patterned correspondence between caudate glucose uptake and neuroactivity to reward and inhibition, showing that the secretin-induced neurometabolic coupling patterns promoted satiation.

**Conclusion:**

These findings suggest that secretin may modulate the BAT-brain metabolic crosstalk and subsequently the neurometabolic coupling to induce satiation. The study advances our understanding of the secretin signaling in motivated eating behavior and highlights the potential role of secretin in treating eating disorders and obesity.

**Trial registration:**

EudraCT no. 2016-002373-35, registered 2 June 2016; Clinical Trials no. NCT03290846, registered 25 September 2017.

**Supplementary Information:**

The online version contains supplementary material available at 10.1007/s00259-023-06124-4.

## Introduction

Secretin is the first hormone ever discovered, and its best-known effect is the induction of pancreatic exocrine secretion [1]. It is secreted while having a meal and has recently been found to induce satiation in both mice and humans [2, 3]. It is suggested that secretin-induced satiation may occur by activation of POMCergic neurons in the medio-basal hypothalamus, thus targeting homeostatic circuits involved in the regulation of food intake [2, 4, 5]. According to the thermoregulatory feeding theory [6], thermogenesis in the brown adipose tissue (BAT) is detected by hypothalamic thermo-sensors modulating central regulation of food intake [2]. Indeed, satiation induced by secretin depends on the activation of BAT thermogenesis where secretin binds to its widely expressed receptors in BAT to activate thermogenesis [2]. This delivers a satiation-stimulating signal to the brain, which may be conveyed by heat-mediated BAT-brain metabolic crosstalk, as suggested previously, but the involvement of endocrine and neuronal communication cannot be excluded [2]. The signaling mode(s) between BAT and brain as well as the targeted brain regions remain to be elucidated in more detail [7].

As the sympathetic nerves controlling BAT thermogenesis harbor afferent sensory fibers projecting to the brainstem, the midbrain, and the forebrain [8], secretin-induced BAT activation may trigger afferent neuronal communication with multiple brain regions. We have previously shown in humans that secretin quenches brain reward-related BOLD responses to appetizing food and increases BAT glucose consumption [3]. The observed neuroanatomical localization of BOLD responses further suggests modulation of the limbic reward system and cognitive control in secretin-induced satiation. In the meanwhile, altered brain glucose metabolism has been linked with binge eating behavior. For instance, our prior studies show that caudate glucose uptake (GU) is upregulated in obese subjects, evidenced by [^18^F]FDG PET measures during hyper-insulinemic clamp [9, 10]. Following bariatric surgery, however, caudate GU is decreased. Hence, it is justified to probe whether secretin, via the BAT-brain axis, further affects the brain glucose metabolism and consequently the neurometabolic coupling with cerebral BOLD signals associative to satiation.

Satiation is linked with altered patterns of neural activations in the cortex [3, 11]. Our previous study especially shows that secretin-mediated satiation delivers a suppressive effect on brain BOLD responses to appetizing food cues. Conversely, elevated neural activation to food cues has been shown to be increased in obese subjects [9], suggesting a shared neural basis for secretin-induced satiation and trait-level eating behavior. While we have shown that secretin downregulates reward-related neural BOLD responses, it is still unclear whether this effect is simply due to reduced sensitivity to visual food stimuli, or whether it is also accompanied by increased cognitive control that suppresses a motivation to eat. Aberrant brain activity in response inhibition has been closely linked with trait-level binge eating [12, 13]. Therefore, it is possible that secretin leads to satiation also via modulating brain inhibitory control in healthy subjects. However, this remains to be explored.

Brain BOLD activity is tightly linked with regional brain glucose metabolism at both resting state [14, 15] and during tasks, yet with markedly different patterns of association [16]. More specifically, neurometabolic coupling during cognitive tasks shows dissociations between the two dimensions of measures, especially for negative BOLD responses. Neuroimaging of satiation and fasting states suggests varying cortical BOLD responses [3, 11], and therefore, neurometabolic coupling between cortical BOLD and metabolic supply to the central reward hub may possess varying patterns. Decoding the secretin-mediated neurometabolic coupling patterns may reveal novel brain mechanism of the motivated eating behavior. However, to our knowledge, no previous studies have investigated these aspects.

In the current study, we specifically investigated the central mechanism of secretin mediated satiation, extending our placebo controlled GUTBAT Trial study [3]. We first investigated whether the secretin-mediated BAT glucose uptake was correlated with corresponding brain glucose update, determining whether secretin modulates brain glucose metabolism through the BAT-brain axis. We then studied whether secretin modulates brain inhibitory control by measuring cortical BOLD signals during a response inhibition task. Finally, we examined whether the secretin-sensitive metabolic supply is directly linked with the secretin-modulated brain inhibitory function and food reward responses, using PET-fMRI fusion analysis. We hypothesize that secretin modulates glucose metabolism in central reward hubs through the BAT-brain axis. We further hypothesize that the secretin-sensitive metabolic supply, via gearing neurometabolic coupling, affects cognitive control and reward processing to cause satiation.

## Methods

### Study design

**Fig. 1:**
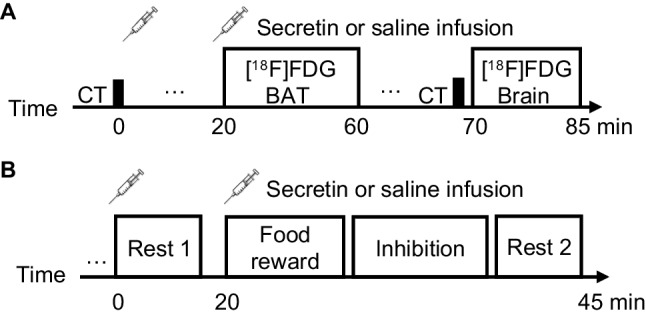
Design of the study. (**A**) Timeline of the CT and [^18^F]FDG PET measures. (**B**) Timeline of the fMRI measures during behavioral tasks or resting states

We investigated the effects of intravenous secretin infusions on BAT and brain metabolism (measured with [^18^F]FDG PET), especially regarding the metabolic crosstalk between BAT and brain. We also studied the brain BOLD responses to inhibitory control (measured with fMRI), extending our previous report on the effect of secretin infusion on food-reward responses [3]. This randomized crossover study was placebo-controlled (Fig. 1), and the participants were blinded to the intervention. All scans were performed in the morning after an overnight fast and at room temperature. Repeated PET measures during placebo and secretin conditions had an interval of 2–30 days (median 15 days) and fMRI 7–30 days (median 14.5 days). The study protocol was reviewed and approved by the Ethics Committee of the Hospital District of Southwest Finland. The PET/CT trial was prospectively registered in the EudraCT registry 2.6.2016 (EudraCT Number: 2016–002,373-35) and a major amendment which included the fMRI study was registered prospectively in Clinical Trials registry 25.9.2017 (Clinical Trials no. NCT03290846).

### Subjects

Fifteen healthy male participants (mean (s.d.) age 41.6 ± 12.1 years, BMI 23.6 ± 1.9 kg/m^−2^) took part in the [^18^F]FDG PET brain imaging study. In parallel, fourteen healthy male participants (age 34.4 ± 14.6 years, BMI 23.3 ± 1.8 kg/m^−2^) joined the fMRI study. Among these participants, a total of 10 males were studied with both [^18^F]FDG PET and fMRI. Lean subjects were recruited in the study, since overweight subjects typically have less active BAT. The experiment was conducted according to the declaration of Helsinki and all participants provided written informed consent for participating in the study (Clinical Trials no NTC03290846).

### BAT PET data acquisition and processing

The [^18^F]FDG PET scans were conducted using GE Discovery (GE DiscoveryTM ST System, General Electric Systems) as described previously [3]. First, a CT scan of the neck was performed for anatomic localization. Next, 150 MBq of [^18^F]FDG was administered for measuring GU [17] and a second two minute infusion of placebo or secretin was initiated. Dynamic 40 min scanning was started simultaneously on the neck region (frames: 1 × 1 min, 6 × 30 s, 1 × 1 min, 3 × 5 min, and 2 × 10 min). Arterialized venous plasma radioactivity samples were collected during the scan by heating the arm from which blood samples were drawn, as described previously [18]. Radiotracer [^18^F]FDG was produced using FASTlab synthesis platform (GE Healthcare) as previously described [19].

Image analysis was conducted with Carimas 2.8 software (Turku PET Center, Turku, Finland). Regions of interest (ROI) were manually outlined in the fusion images, composed of the dynamic [^18^F]FDG PET image and the corresponding CT image. To analyze BAT GU, ROIs were drawn on the supraclavicular fat depots including only voxels with CT Hounsfield Units (HU) within the adipose tissue range (-50 to -250 HU) [20]. For tissue glucose uptake calculations, time activity curves (TAC) were generated for the ROIs. Regional TAC data was analyzed by taking into account the radioactivity in arterialized plasma using the Patlak model [21]. A lumped constant value of 1.14 was used for adipose tissue [22].

### Brain PET data acquisition and processing

Dynamic 15 min brain PET scans (frames: 3 × 5 min) were started 70 min after [^18^F]FDG injection. Head movement was prevented by strapping to the scan table. Computed tomography scans were obtained prior to the PET scans for attenuation correction and the T1-weighted MR images (TR 8.1 ms, TE 3.7 ms, flip angle 7°, 256 × 56 × 176 mm^3^ FOV, 1 × 1 × 1 mm^3^ voxel size) were taken using the 3-Tesla Philips Ingenuity PET/MR scanner for anatomical normalization and reference. PET data were preprocessed by the automatic pipeline Magia [23]. PET brain images were motion-corrected and then co-registered to the corresponding structural MR images. Brain glucose uptake was estimated using fractional uptake rate calculated as a ratio between tissue activity at time *T* and integral of plasma activity from time 0 to *T* [24]; all frames were included.

### Inhibitory control task

**Fig. 2:**
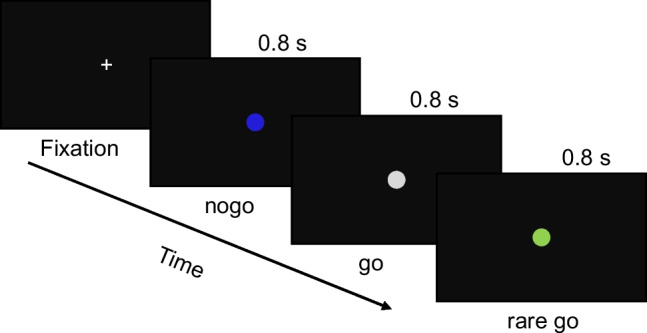
fMRI paradigm for the response inhibition task. Participants were instructed to press the button on the go and rare go signals and refrain from pressing the button on the no go signals

Participants were instructed to press a button using the left hand when it was a go signal or to withhold from pressing the button when it was a no go signal (Fig. 2), while brain haemodynamic responses were measured. This task was performed immediately after the anticipatory food-reward task (supplementary Fig. S1 and supplementary method). Small dots were presented one-by-one in the middle of the computer screen, with an interval of 0.8 s. The dots could be either gray (70% of all cases), green (15% of all cases), or blue (15% of all cases). The gray dots were always “go” signal prompting the subject to press the button. The blue and green dots were randomly assigned to either rare “go” or “no go” signal for each participant. The statistical contrast for inhibitory activation by either blue or green dots occurred with equal probability. Stimulus delivery was controlled by the presentation software (Neurobehavioral System, Inc., Berkeley, CA, USA).

### fMRI data acquisition and processing

The Phillips Ingenuity TF PET/MR 3 T whole-body scanner was used to collect MRI data. Structural brain images with resolution of 1 mm^3^ were acquired using a T1-weighted sequence (TR 9.8 ms, TE 4.6 ms, flip angle 7°, 250 mm FOV, 256 × 256 reconstruction matrix). Functional MRI data were acquired using a T2*-weighted echo-planar imaging sequence (TR = 2600 ms, TE = 30 ms, 75° flip angle, 240 mm FOV, 80 × 80 reconstruction matrix, 62.5 kHz bandwidth, 3.0 mm slice thickness, 45 interleaved slices acquired in ascending order without gaps). A total of 145 functional volumes were acquired during the inhibitory control task. A total of 165 functional volumes were acquired during the food-reward task, see [3].

MRI data were processed using the fMRIPrep 1.3.0.2 [25]. Structural T1 images were processed following steps: correction for intensity non-uniformity, skull-stripping, brain surface reconstruction, and spatial normalization to the ICBM 152 Nonlinear Asymmetrical template version 2009c [26] using nonlinear registration with antsRegistration (ANTs 2.2.0) and brain tissue segmentation. Functional MRI data were processed in following steps: co-registration to the T1 reference image, slice-time correction, spatial smoothing with a 6 mm Gaussian kernel, automatic removal of motion artifacts using ICA-AROMA [27], and resampling to the MNI152NLin2009cAsym standard space. Quality of images was inspected visually for the whole-brain field of view coverage, proper alignment to the anatomical images, and signal artifacts, and inspected also via the visual reports of fMRIPrep. We set to exclude images having large movement artifacts with more than 25% of the volumes exceeding 0.5-mm framewise displacement [28], and accordingly, all functional data were included in the current study.

### Statistical analysis

#### PET data

The full-volume brain data were analyzed using SPM12 (Wellcome Trust Center for Imaging, London, UK; http://www.fil.ion.ucl.ac.uk/spm). First, paired-*T* test (secretin vs. placebo conditions) was used to examine the effect of secretin on brain GU. Second, paired-*T* test was done while controlling for covariates of BAT GU, where the between-condition contrast essentially indicated an interaction effect between Condition (placebo vs. secretin) and the covariates. This was used to evaluate the modulatory effect of secretin on the BAT-brain metabolic crosstalk regarding GU. Considering the dependency between Condition and BAT GU, statistical threshold was set at *p* < 0.001 with FDR cluster level correction (n.b., we kept voxel-level primary threshold and cluster-level threshold the same in all analysis) to minimize potential false positive findings. Next, GLMs using BAT GU as regressor, separately for placebo and secretin conditions, were constructed to examine the correlation between BAT GU and brain GU. Statistical threshold was set at *p* < 0.05 with FDR cluster level correction. In addition, correlation between BAT GU and caudate GU at caudate was done using Kendall correlation test using R statistical software (version 3.6.3). Caudate GU was estimated using MarsBarR toolbox [29] based on the ROI defined by the AAL atlas [30].

#### fMRI data analysis

Reaction times for the go trials (either including or excluding rare go trials) were analyzed and those below 100 ms or over 800 ms were excluded. Accuracy rates were estimated as the percentage of “no response” in all no go trials. RTs and accuracy rates were analyzed separately using mixed effect linear model with condition as the fixed factor and subject as random factor. All analysis were done using R statistical software.

The full-volume fMRI data were analyzed in SPM12. The whole-brain random effects model was applied using a two-stage process with separate first and second levels. For each subject, first-level GLM was used to predict regional effects of task parameters (no go vs. go signals; go signals including both “go” and “rare go” signals) on BOLD indices of activation and data from both conditions (secretin vs. placebo) were fitted into the same model. Statistical threshold was set at *p* < 0.05 with FDR correction at cluster level.

ROIs including the insula and motor area (including the pre-supplementary motor area, pre- and post-central cortex) were selected based on previously validated involvement in response inhibition, for example, [31]. ROI values were estimated using MarsBarR toolbox based on the ROI defined by the AAL atlas. ROI data were estimated and fitted to linear regression models using condition, trial type (go vs. no go), and an interaction between condition and trial types, as factors.

#### PET-fMRI fusion analysis

First-level BOLD contrast images were firstly analyzed using paired-*T* test with caudate GU as covariate. Later, GLMs using caudate GU as regressors, separately for placebo and secretin conditions, were constructed to examine the correlation between caudate GU and cerebral BOLD signal during either inhibitory or food-reward responses. Statistical threshold was set at *p* < 0.05, FDR corrected at cluster level.

## Results

### Effect of secretin on BAT and brain GU metabolic crosstalk

**Fig. 3:**
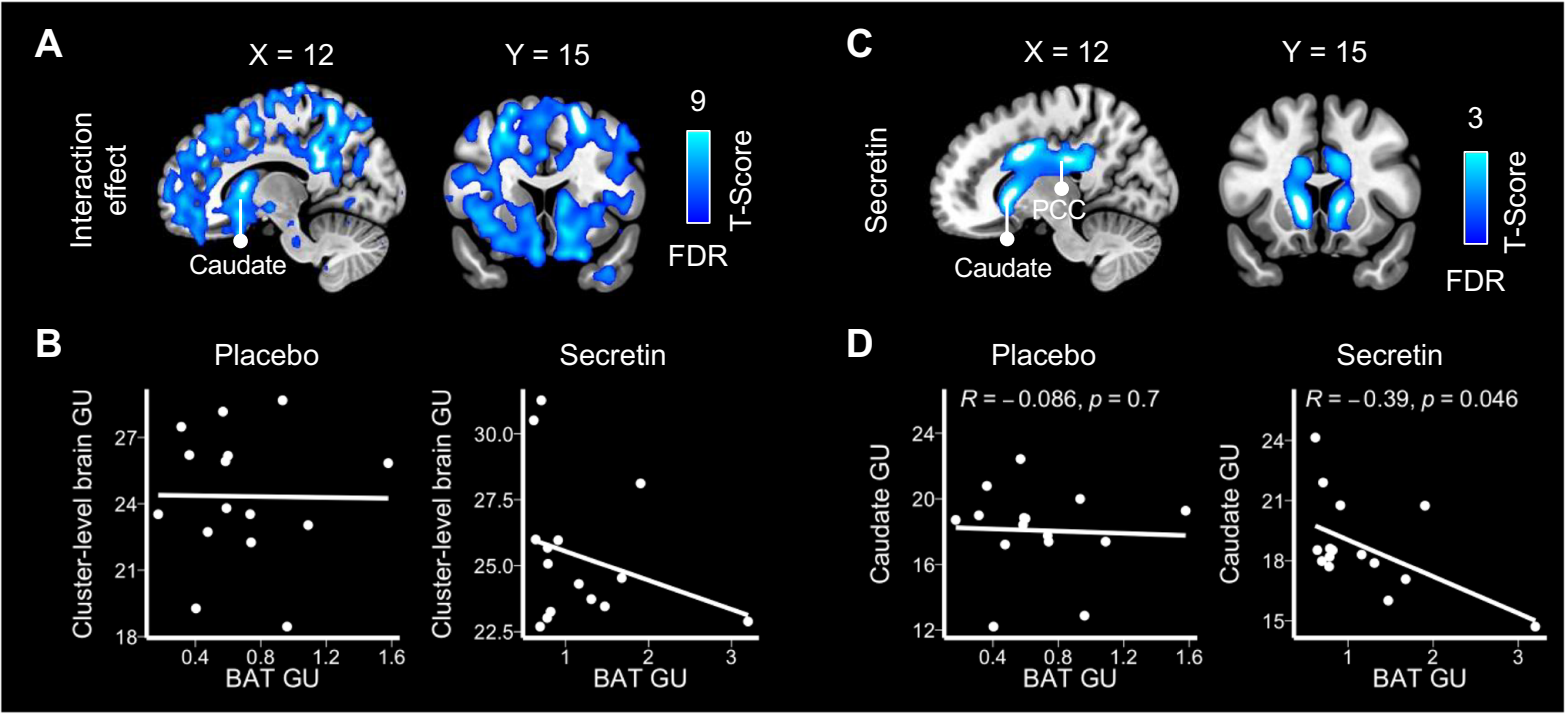
Secretin modulated the BAT-brain metabolic crosstalk (*n* = 15). (**A**) Brain regions where secretin impacts BAT GU and brain GU associations. This was illustrated by a widespread effect of condition (secretin vs. placebo) on brain GU while controlling for BAT GU. Data were thresholded at *p* < 0.001 with FDR cluster-level correction. (**B**) Brain cluster-level (one large cluster of 72,861 voxels) GU values were plotted to corresponding BAT GU values for visualization. (**C**) BAT GU was a significant predictor for GU in the brain caudate and cingulate cortex in the secretin condition, but not in any brain area in the placebo condition. Data were thresholded at *p* < 0.05 with FDR cluster-level correction. (**D**) ROI analysis showed that GU at caudate and BAT were negatively correlated during the secretin condition but not in the placebo condition. BAT, brown adipose tissue; GU, glucose uptake; PCC, post-cingulate cortex. GU is expressed as μmol*100 g^−1^*min^−1^

Full-volume analysis of brain GU levels revealed no statistically significant difference between the placebo and secretin conditions. In contrast, there was a significant difference between conditions while controlling for BAT GU (Fig. 3A and B). This may highlight a widespread interference of secretin on the BAT and brain metabolic crosstalk. Even though one participant had relatively higher BAT GU during the secretin condition compared to the other participants, excluding this participant from the analyses did not affect the result (supplementary Fig. S2).

Further analysis revealed that BAT GU was a significant predictor for GU in the caudate and cingulate area in the secretin condition (Fig. 3C), but not a significant predictor for GU in any brain area in the placebo condition. Also, ROI level analysis revealed that caudate GU was negatively correlated with BAT GU in the secretin but not placebo condition (Fig. 3D). Cingulate GU was not significantly correlated with BAT GU in either condition (supplementary Fig. S3).

### Effect of secretin on inhibitory neural responses

Compared to placebo condition, secretin condition was associated with faster reaction speed in Go trials while including both “go” or “rare go” trials as go trials in the analysis (*β* = -0.03, 95% CI [-0.05, -0.01]). When the “rare go” trails were excluded, secretin was similarly associated with faster reaction speed (*β* = -0.02, 95% CI [-0.04, -0.0004]). There was no secretin-dependent effect on accuracy of responses (*β* = -0.06, 95% CI [-0.13, 0.02]) between the two conditions.

**Fig. 4:**
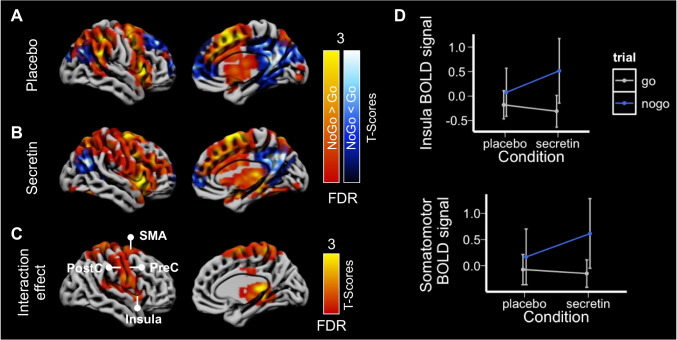
Secretin modulated the inhibition related neural activity (*n* = 14). Contrast images demonstrated the increased and decreased neural activity during inhibition in (**A**) the placebo condition and (**B**) the secretin condition. (**C**) Interaction contrast between type of trials (no go vs. go trials) and condition (placebo vs. secretion) showed the modulatory effect of secretin on inhibition-related neural activity. Data were thresholded at *p* < 0.05 with FDR cluster-level correction and right hemispheres are presented for illustration. (**D**) ROI analysis showed an increased neural activity during inhibition in the motor area (comprising the supplementary motor area, precentral, and postcentral gyri) and insula in the secretin condition. Error bars represent 95% confidence interval. BOLD, blood oxygen level–dependent; SMA, supplementary motor area; PostC, postcentral cortex; PreC, precentral cortex

Brain BOLD signals in no go versus go trials were associated with elevated activity in insula, supplementary motor area, precentral gyrus, postcentral gyrus, anterior and middle cingulate cortex, precuneus, and thalamus in the placebo conditions (Fig. 4A). In the secretin condition, elevated activity in these brain regions was enhanced (Fig. 4B), as was also confirmed by an interaction effect between condition and type of trials (no go vs. go; Fig. 4C). The ROI analysis yielded corroborating findings (Fig. 4D). While no significant interaction effects were found between condition and type of trials, in the secretin condition, no go trials were associated with significantly increased BOLD signal in sensory and motor area (beta = 0.76, 95% CI [0.08, 1.45], *p* = 0.03) and insula (beta = 0.83, 95% CI [0.13, 1.52], *p* = 0.02). No similar effects were found in the placebo condition.

Meanwhile, dampened activity was found in lateral and medial prefrontal cortex and posterior cingulate area in both conditions (Fig. 3A and B).

Our findings so far have revealed that secretin (i) stimulates metabolic crosstalk between BAT and caudate, (ii) increases brain BOLD signals in inhibitory control, and (ii) reduces BOLD response to appetizing food pictures (supplementary Fig. S4 and [3]). Next, we investigated whether these effects are tightly linked, by studying possible secretin-dependent brain neurometabolic coupling. In the following session, we reported (i) whether secretin modulated correspondence between caudate GU and inhibitory BOLD responses and then (ii) whether secretin modulated correspondence between caudate GU and reward-related BOLD responses.

### Correspondence between caudate glucose uptake and inhibitory responses

**Fig. 5:**
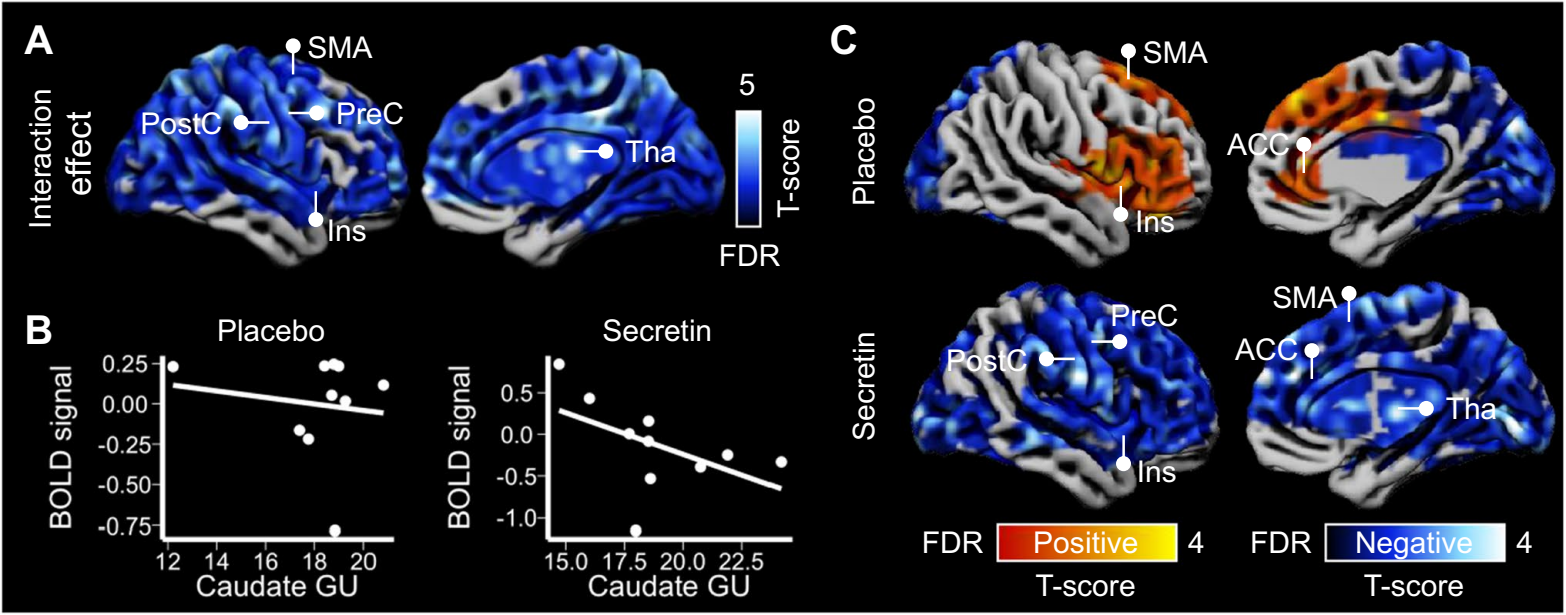
Secretin modulated the correspondence between caudate GU and cerebral BOLD during inhibition (*n* = 10). (**A**) There was a widespread effect of condition on BOLD signals during inhibition while controlling for caudate GU. (**B**) Plots of cluster-level (one large cluster of 44,150 voxels) BOLD signal to the corresponding caudate GU in different conditions for visualization. (**C**) In the placebo condition, there were both positive and negative associations between caudate GU and cerebral BOLD during inhibition, while in the secretin condition, there was only negative association. Data were thresholded at *p* < 0.05 with FDR cluster-level correction. SMA, supplementary motor area; PreC, precentral cortex; PostC, postcentral cortex; ACC, anterior cingulate cortex; Ins, insula; Tha, thalamus. GU is expressed as μmol*100 g^−1^*min^−1^. All statistical parametric images can be found from NeuroVault at https://neurovault.org/collections/WGTKYETH/

First-level BOLD contrast images (no go vs. go) were analyzed using paired-*T* test with caudate GU as covariate. There was a widespread effect of condition on inhibition-related BOLD responses while controlling for caudate GU (Fig. 5A and B). In the separate analysis for each condition, individual-specific caudate GU was used as predictor for corresponding BOLD contrast image. In the placebo condition, caudate GU levels were associated with increased neural activity in the lateral prefrontal cortex, anterior- and mid-cingulate, and insula; also, caudate GU levels were associated with reduced activity in the parietal and occipital areas (Fig. 5C & supplementary Fig. S5). In the secretin condition, caudate GU levels were associated with globally reduced BOLD activities.

### Correspondence between caudate glucose uptake and neural activity during reward response

**Fig. 6:**
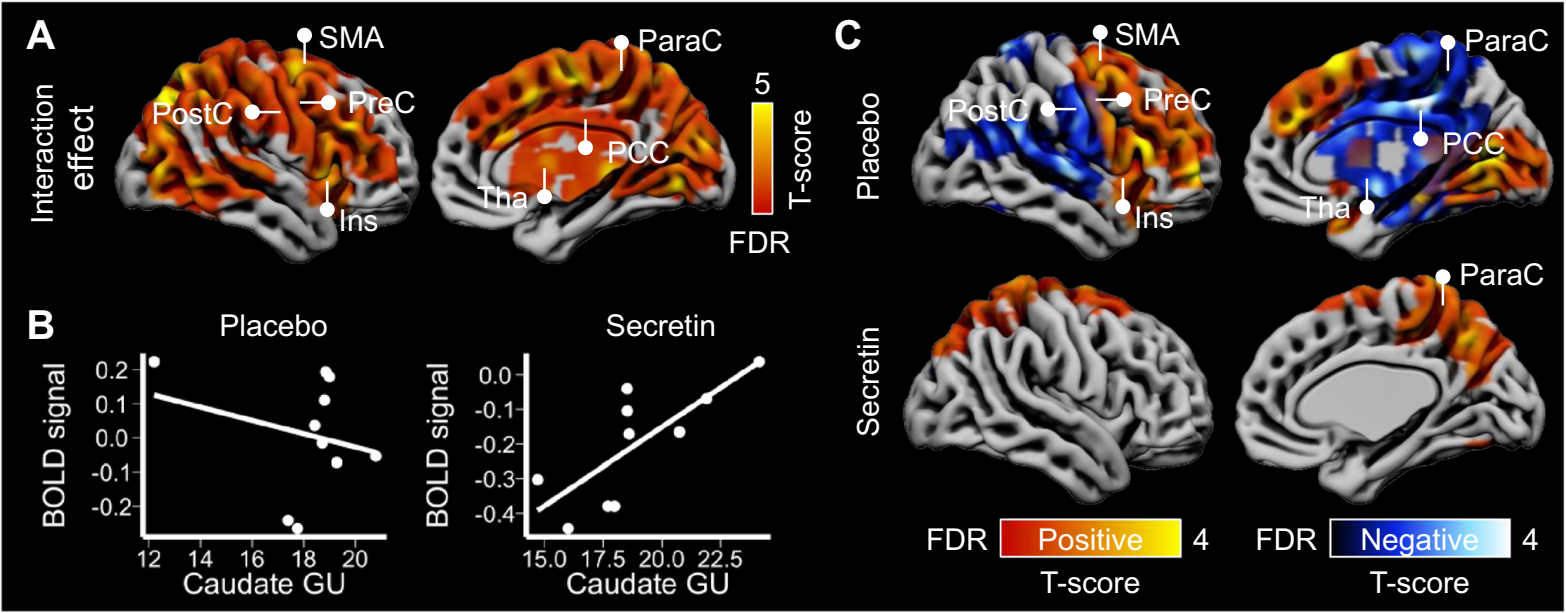
Secretin modulated the correspondence between caudate GU and cerebral BOLD signals during food-reward responses (*n* = 10). (**A**) There was a widespread effect of condition on BOLD signals during reward response while controlling for caudate GU. (**B**) Plots of cluster-level (one large cluster of 34,187 voxels) BOLD signal to the corresponding caudate GU in different conditions for visualization. (**C**) In the placebo condition, there were both positive and negative associations between caudate GU and cerebral BOLD signals in reward response, while in the secretin condition, there was only a positive association. Data were thresholded at *p* < 0.05 with FDR cluster-level correction. ParaC, paracentral gyrus; SMA, supplementary motor area; PreC, precentral cortex; PostC, postcentral cortex; PCC, posterior cingulate cortex; Ins, insula; Tha, thalamus. GU is expressed as μmol*100 g^−1^*min^−1^

Similarly, first-level BOLD contrast images (appetizing vs. bland food) were analyzed using condition and caudate GU as predictors. There was a widespread effect of condition on reward-related BOLD responses while controlling for caudate GU (Fig. 6A and B). In the placebo condition, caudate GU levels were associated with increased neural activity in the lateral prefrontal cortex, insula and occipital cortex; also, they were associated with reduced activity in the post-central and parietal areas (Fig. 6C; supplementary Fig. S6). In the secretin condition, caudate GU levels were only associated with increased BOLD activity in brain regions including the para-central area, while those negative associations disappeared.

## Discussion

**Fig. 7:**
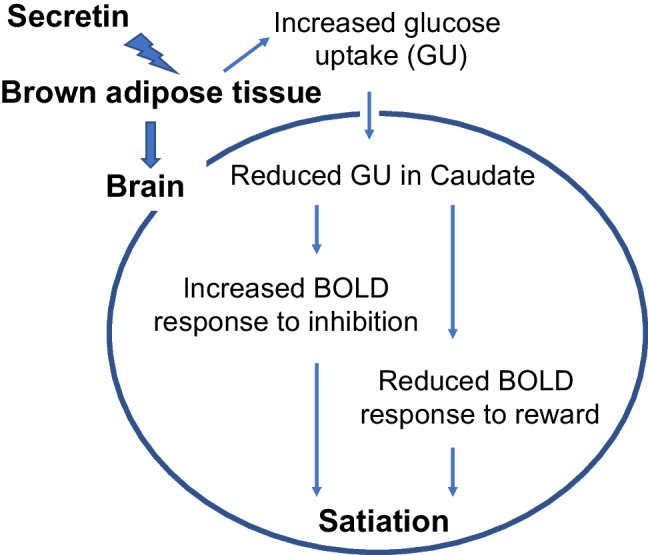
Mechanism of the secretin-induced satiation. BOLD, blood-oxygen-level-dependent signal

The current study reveals in vivo that secretin induces metabolic BAT-brain crosstalk, as indicated by the negatively correlated glucose uptake rates after secretin infusion. Using functional measures of neuroactivity, our study also shows that secretin directly enhances inhibitory control, extending our previous report that secretin downregulates of brain responses to appetizing food images and causes satiation [3]. Besides, the PET-fMRI fusion data analysis reveals the secretin-specific patterns of neurometabolic coupling, during both reward responses and inhibition, further highlighting the role of secretin modulated BAT-brain axis in motivated eating behavior. Taken together, we propose that secretin modulates BAT-brain metabolic crosstalk and subsequently shapes neurometabolic coupling to promote satiation (Fig. 7). This study uncovers a potential brain mechanism for secretin-induced satiation, bearing prospective clinical significance in dealing with eating disorders.

Our previous studies have highlighted that BAT has a dual role in maintaining energy homeostasis [2, 3]. It is not merely a heater organ that increases energy expenditure but also regulates energy intake. In both mice and men, feeding activates BAT, but the concept of thermoregulatory feeding has not been investigated in detail, especially in humans. Our previous study has introduced a neurobiological concept for heat induced satiation in mice [2]. Since detailed methods used in mouse models are not fully translatable to humans, the question whether BAT conveys its satiation effect to the central nervous system through heat or other means, has remained unresolved. Here, our results show that secretin induces metabolic crosstalk between BAT and brain to promote satiation. While this finding further supports the significance of a gut-BAT-brain axis in controlling eating behavior, it highlights the complexity of brain mechanisms under this secretin signaling pathway.

The role of the caudate in reward-oriented action has been well established [32, 33]. Here, our findings showed a link between secretin-stimulated BAT glucose uptake and the downregulation of caudate metabolism. With [^18^F]FDG PET measures, we showed that secretin induced higher BAT glucose uptake, which may subsequently stimulated lower caudate glucose uptake. Our fusion analysis data further indicated that lower caudate glucose supply is associated with lower BOLD response to rewarding food images (i.e., enhanced positive correlation) and higher BOLD response in inhibition (i.e., enhanced negative correlation**)**. In contrast, these types of correspondence demonstrate largely varied patterns during the placebo condition.

Secretin’s suppressive effect on acute brain responses to appetizing food images and enhancement of cognitive control further highlights its potential role in weight control. Previous studies have shown that binge eating behavior is associated with enhanced BOLD response to rewarding food images [9] and aberrant inhibitory control [12, 13]. Here, secretin seems to affect both these cognitive functions, along with trait-level responses such as reduced motivation to eat, as reported in our previous study [3]. Furthermore, both these cognitive responses are directly linked with the secretin-mediated change in caudate glucose uptake levels, suggesting that secretin induces satiation most probably via modulating the neurometabolic coupling between caudate glucose supply and cerebral BOLD responses.

How this metabolic crosstalk between BAT and caudate occurs remains elusive. BAT is largely innervated by the central nervous system [8, 34], probably including the caudate as supported by findings through the anterograde transneuronal viral tract tracing [35]. Our data showing that secretin activates BAT thermogenesis and metabolic crosstalk with the caudate suggests a potential feedback loop in the BAT-brain axis. The negative correlation between BAT and caudate GU may complement the thermoregulatory feeding theory [2, 6], revealing that increased thermogenesis in BAT is detected by the brain leading to restricted glucose consumption in caudate. Conversely, central control of BAT activity via neurotransmitters has been also reported. For example, release of noradrenaline activates BAT adrenergic receptors thus to stimulate biochemical reactions in mitochondria and thermogenesis [8]. Also, intravenous and intracerebroventricular administration of fentanyl enhances BAT sympathetic nerve activity and thermogenesis [36], supporting a potential role of central opioid signaling in BAT thermogenesis [37]. However, whether these neurotransmission signaling pathways are involved in secretin-mediated BAT-caudate crosstalk, subsequently associative to satiation, remains to be explored.

We have previously shown that human BAT has high expression of secretin receptors and that secretin infusion enhances thermogenesis [3]. On the other hand, secretin is a relatively large peptide hormone (~ 3000 Da), but it has been previously shown that it can cross the blood–brain barrier (BBB) in young rats [38]. Though evidence of secretin passing the BBB in humans is currently lacking, this cannot be excluded. Still, the present results may be largely explained through a brain-BAT axis, considering those secretin-mediated correspondences between caudate GU and cerebral BOLD responses to both reward and inhibition. Importantly, secretin infusion did not affect caudate GU directly. Taken together, this data suggests the presence of a functional BAT-brain axis as involved in the neural control of satiation.

## Limitations

Despite showing a significant correlation between BAT and caudate glucose uptake under secretin administration, a causal link between the effects cannot be shown with the implemented method. Furthermore, considering the complexity of PET-fMRI instrumentation, we involved only male participants and the number of studied subjects, especially for the fusion analysis, was small; for fMRI data analysis and fusion analysis, we used FDR-corrected cluster-level *p* value 0.05. Brain scans were all taken at around 70 min after injection of radiotracer. Although such acquisition protocol is common and k4 needs to be controlled for only after 120 min, it is still possible that k4 might be an issue already at around 70 min for [^18^F]FDG PET scans.

## Conclusions

The current study established a secretin-mediated BAT-caudate axis in regulating the motivated eating behavior in humans. Both metabolic and cognitive level evidence suggests that secretin may be a potential drug for the treatment of eating disorders such as obesity.

## Supplementary Information

Below is the link to the electronic supplementary material.

Supplementary file1 (DOCX 3.81 MB)

## Data Availability

The current study is based on human subject PET– fMRI data. As per Finnish legislation, the medical imaging data are considered sensitive personal information and cannot be publicly shared even in anonymized format. Enquiries regarding the dataset can be sent to Pirjo Nuutila by: email to pirjo.nuutila@utu.fi or post to Turku PET Centre c/o Turku University Hospital, Kiinamyllynkatu 4-8, FI-20520 Turku, Finland.

## References

[1] Bayliss WM, Starling EH. The mechanism of pancreatic secretion. J Physiol. 1902. 10.1113/jphysiol.1902.sp000920 PMC1540572 16992627

[2] Li Y, Schnabl K, Gabler SM, Willershäuser M, Reber J, Karlas A (2018). Secretin-activated brown fat mediates prandial thermogenesis to induce satiation. Cell.

[3] Laurila S, Sun L, Lahesmaa M, Schnabl K, Laitinen K, Klén R (2021). Secretin activates brown fat and induces satiation. Nat Metab.

[4] Jeong JH, Lee DK, Liu SM, Chua SC, Schwartz GJ, Jo YH. Activation of temperature-sensitive TRPV1-like receptors in ARC POMC neurons reduces food intake. PLoS Biol. 2018. 10.1371/journal.pbio.2004399 PMC5915833 29689050

[5] Cheng CYY, Chu JYS, Chow BKC. Central and peripheral administration of secretin inhibits food intake in mice through the activation of the melanocortin system. Neuropsychopharmacology. 2011. 10.1038/npp.2010.178 PMC3055665 20927047

[6] Himms-Hagen J. Role of brown adipose tissue thermogenesis in control of thermoregulatory feeding in rats: a new hypothesis that links thermostatic and glucostatic hypotheses for control of food intake. Proc Soc Exp Biol Med. 1995; 10.3181/00379727-208-43847a 7831348

[7] Schnabl K, Li Y, U-Din M, Klingenspor M. Secretin as a satiation whisperer with the potential to turn into an obesity-curbing knight. Endocrinol. (United States). 2021. 10.1210/endocr/bqab113 34089599

[8] Bartness TJ, Vaughan CH, Song CK (2010). Sympathetic and sensory innervation of brown adipose tissue. Int J Obes.

[9] Nummenmaa L, Hirvonen J, Hannukainen JC, Immonen H, Lindroos MM, Salminen P, et al. Dorsal striatum and its limbic connectivity mediate abnormal anticipatory reward processing in obesity. PLoS One. 2012;7. 10.1371/journal.pone.0031089 PMC3272045 22319604

[10] Tuulari JJ, Karlsson HK, Hirvonen J, Hannukainen JC, Bucci M, Helmiö M (2013). Weight loss after bariatric surgery reverses insulin-induced increases in brain glucose metabolism of the morbidly obese. Diabetes.

[11] Thomas JM, Higgs S, Dourish CT, Hansen PC, Harmer CJ, McCabe C. Satiation attenuates BOLD activity in brain regions involved in reward and increases activity in dorsolateral prefrontal cortex: an fMRI study in healthy volunteers. Am J Clin Nutr. 2015. 10.3945/ajcn.114.097543 25833968

[12] Lock J, Garrett A, Beenhakker J, Reiss AL. Aberrant brain activation during a response inhibition task in adolescent eating disorder subtypes. Am J Psychiatry. 2011. 10.1176/appi.ajp.2010.10010056 PMC3016457 21123315

[13] Hege MA, Stingl KT, Kullmann S, Schag K, Giel KE, Zipfel S, et al. Attentional impulsivity in binge eating disorder modulates response inhibition performance and frontal brain networks. Int J Obes. 2015. 10.1038/ijo.2014.99 24909828

[14] Aiello M, Salvatore E, Cachia A, Pappatà S, Cavaliere C, Prinster A, et al. Relationship between simultaneously acquired resting-state regional cerebral glucose metabolism and functional MRI: A PET/MR hybrid scanner study. Neuroimage. 2015. 10.1016/j.neuroimage.2015.03.017 25791784

[15] Tomasi D, Wang GJ, Volkow ND. Energetic cost of brain functional connectivity. Proc Natl Acad Sci U S A. 2013. 10.1073/pnas.1303346110 PMC3746878 23898179

[16] Stiernman LJ, Grill F, Hahn A, Rischka L, Lanzenberger R, Lundmark VP, et al. Dissociations between glucose metabolism and blood oxygenation in the human default mode network revealed by simultaneous PET-fMRI. Proc Natl Acad Sci U S A. 2021;118. 10.1073/pnas.2021913118 PMC8271663 34193521

[17] Orava J, Nuutila P, Lidell ME, Oikonen V, Noponen T, Viljanen T (2011). Different metabolic responses of human brown adipose tissue to activation by cold and insulin. Cell Metab.

[18] Lahesmaa M, Orava J, Schalin-Jäntti C, Soinio M, Hannukainen JC, Noponen T, et al. Hyperthyroidism increases brown fat metabolism in humans. J Clin Endocrinol Metab. 2014. 10.1210/jc.2013-2312 24152690

[19] Hamacher K, Coenen HH, Stocklin G. Efficient stereospecific synthesis of no-carrier-added 2-[18F]-fluoro-2-deoxy-D-glucose using aminopolyether supported nucleophilic substitution. J Nucl Med. 1986. 3712040

[20] Din MU, Raiko J, Saari T, Saunavaara V, Kudomi N, Solin O (2017). Human brown fat radiodensity indicates underlying tissue composition and systemic metabolic health. J Clin Endocrinol Metab.

[21] Patlak CS, Blasberg RG. Graphical evaluation of blood-to-brain transfer constants from multiple-time uptake data. Generalizations. J Cereb Blood Flow Metab. SAGE PublicationsSage UK: London, England; 1985;5:584–90. 10.1038/jcbfm.1985.87 4055928

[22] Virtanen KA, Peltoniemi P, Marjamäki P, Asola M, Strindberg L, Parkkola R (2001). Human adipose tissue glucose uptake determined using [18F]-fluoro-deoxy-glucose ([18F]FDG) and PET in combination with microdialysis. Diabetologia Springer-Verlag.

[23] Karjalainen T, Tuisku J, Santavirta S, Kantonen T, Bucci M, Tuominen L, et al. Magia: robust automated image processing and kinetic modeling toolbox for PET neuroinformatics. Front Neuroinform [Internet]. 2020;14. Available from: https://www.frontiersin.org/articles/10.3389/fninf.2020.00003/full. 10.3389/fninf.2020.00003 PMC7012016 32116627

[24] Thie JA. Clarification of a fractional uptake concept. J. Nucl. Med. 1995. p. 711–2. 7699475

[25] Esteban O, Markiewicz CJ, Blair RW, Moodie CA, Isik AI, Erramuzpe A (2019). fMRIPrep: a robust preprocessing pipeline for functional MRI. Nat Methods.

[26] Fonov V, Evans A, McKinstry R, Almli C, Collins D (2009). Unbiased nonlinear average age-appropriate brain templates from birth to adulthood. Neuroimage.

[27] Pruim RHR, Mennes M, Buitelaar JK, Beckmann CF (2015). Evaluation of ICA-AROMA and alternative strategies for motion artifact removal in resting state fMRI. Neuroimage.

[28] Parkes L, Fulcher B, Yücel M, Fornito A (2018). An evaluation of the efficacy, reliability, and sensitivity of motion correction strategies for resting-state functional MRI. Neuroimage.

[29] Brett M, Anton J-LL, Valabregue R, Poline J-B. Region of interest analysis using an SPM toolbox - Abstract Presented at the 8th International Conference on Functional Mapping of the Human Brain, June 2–6, 2002, Sendai, Japan. Neuroimage. 2002;16:Abstract 497.

[30] Tzourio-Mazoyer N, Landeau B, Papathanassiou D, Crivello F, Etard O, Delcroix N (2002). Automated anatomical labeling of activations in SPM using a macroscopic anatomical parcellation of the MNI MRI single-subject brain. Neuroimage.

[31] Sebastian A, Pohl MF, Klöppel S, Feige B, Lange T, Stahl C (2013). Disentangling common and specific neural subprocesses of response inhibition. Neuroimage.

[32] Tricomi EM, Delgado MR, Fiez JA. Modulation of caudate activity by action contingency. Neuron. 2004. 10.1016/s0896-6273(03)00848-1 14741108

[33] Hikosaka O, Sakamoto M, Usui S. Functional properties of monkey caudate neurons. III. Activities related to expectation of target and reward. J Neurophysiol. 1989. 10.1152/jn.1989.61.4.814 2723722

[34] Kuruvilla R (2019). Why brown fat has a lot of nerve. Nature.

[35] Vaughan CH, Bartness TJ. Anterograde transneuronal viral tract tracing reveals central sensory circuits from brown fat and sensory denervation alters its thermogenic responses. Am J Physiol - Regul Integr Comp Physiol. 2012. 10.1152/ajpregu.00640.2011 PMC3362143 22378771

[36] Cao WH, Morrison SF (2005). Brown adipose tissue thermogenesis contributes to fentanyl-evoked hyperthermia. Am J Physiol - Regul Integr Comp Physiol.

[37] Sun L, Aarnio R, Herre EA, Kärnä S, Palani S, Virtanen H, et al. [11C]carfentanil PET imaging for studying the peripheral opioid system in vivo: effect of photoperiod on mu-opioid receptor availability in brown adipose tissue. Eur J Nucl Med Mol Imaging. 2022. 10.1007/s00259-022-05969-5 PMC9816189 36166079

[38] Banks WA, Goulet M, Rusche JR, Niehoff ML, Boismenu R. Differential transport of a secretin analog across the blood-brain and blood-cerebrospinal fluid barriers of the mouse. J Pharmacol Exp Ther. 2002. 10.1124/jpet.102.036129 12183664

