# Supplementary material for: Secretin modulates appetite via brown adipose tissue-brain axis
Source: Eur J Nucl Med Mol Imaging. 2023 Feb 11;50(6):1597–606. doi: 10.1007/s00259-023-06124-4 (PMC10119257; doi:10.1007/s00259-023-06124-4)
Supplement: Supplementary file 1 — Supplementary file1 (DOCX 3.81 MB) [file 259_2023_6124_MOESM1_ESM.docx]

**Supplementary data**

**Secretin modulates appetite via brown adipose tissue - brain axis**

Lihua Sun^1,2,3,4*^, Sanna Laurila^3,5,6^, Minna Lahesmaa^3,4^, Eleni Rebelos^3,4^,

Kirsi A. Virtanen^3,4,7^, Katharina Schnabl^8,9,10^, Martin Klingenspor ^8,9,10^,

Lauri Nummenmaa^3,4,11^, Pirjo Nuutila^3,7^

1. Department of Nuclear Medicine, Pudong Hospital, Fudan University, Shanghai, China
2. Department of Nuclear Medicine, Huashan Hospital, Fudan University, Shanghai, China
3. Turku PET Centre, University of Turku, Turku, Finland
4. Turku PET Centre, Turku University Hospital, Turku, Finland
5. Heart Center, Turku University Hospital, Turku, Finland.
6. Department of Medicine, University of Turku, Turku, Finland
7. Department of Endocrinology, Turku University Hospital, Turku, Finland.
8. Chair for Molecular Nutritional Medicine, Technical University of Munich, TUM School of Life Sciences, Freising, Germany
9. EKFZ - Else Kröner Fresenius Center for Nutritional Medicine, Technical University of Munich, Freising, Germany
10. ZIEL – Institute for Food & Health, Technical University of Munich, Freising, Germany
11. Department of Psychology, University of Turku, Finland

*Correspondence to:

Dr. Lihua Sun, Email: sunlihua@fudan.edu.cn

**Methods**

*Anticipatory food reward task*

We used a previously established task protocol for inducing anticipatory reward [1], by showing the participants pictures of palatable (e.g. chocolate, pizza, cakes), and bland (e.g. lentils, cereal, eggs) food pictures (**supplementary Fig. 1**). This task simulates situations where appetite is triggered by anticipating the actual feeding via visual food cues, such as those in advertisements. The pictures were rated in a previous study by independent participants; the ratings showed that the appetizing foods were evaluated more pleasant than the bland foods, t(31) = 4.67, p < 0.001 [2]. During the task, participants viewed alternating 16.2-s epochs with pictures of palatable or non-palatable foods. Each epoch contained nine stimuli from one category, intermixed with fixation crosses. Each food stimulus was presented on either the right or the left side of the screen. Participants were instructed to indicate its location by pressing corresponding buttons, simply to ensure that participants had to pay attention to the stimuli. Stimulus delivery was controlled by the Presentation software (Neurobehavioral System, Inc., Berkeley, CA, USA).

**Supplementary Figure S1.** fMRI paradigm for the anticipatory food reward task. Participants are instructed to press a button to indicate the location (either left or right side of the screen) of each food picture.

**Results**

***Supplementary Figure S2.*** *Interaction effect between Condition (secretin vs. placebo) and BAT GU on the brain GU, when excluding a subject with highest BAT GU (n = 14). Data were thresholded at p<0.001 with FDR cluster-level correction.*

***Supplementary Figure S3.*** *Correlation between cingulate GU and BAT GU. BAT GU was not significant predictor for* ***A)*** *middle cingulate cortex GU or* ***B)*** *posterior cingulate cortex GU in either placebo or secretin condition. BAT = brown adipose tissue. GU is expressed as μmol*100g^-1^*min^-1^.*

***Supplementary Figure S4.*** *Secretin modulated the reward-related neural activity. Contrast images demonstrated the increased neural activity during inhibition in* ***A)*** *the placebo condition and both increased and dampened activity in* ***B)*** *the secretin condition.* ***C)*** *Interaction contrast between Trial types (appetizing vs. bland food images) and Condition (placebo vs. secretion) showed the modulatory effect of secretin on reward-related neural activity. Full-volume analysis, Data were thresholded at p<0.05 with FDR cluster-level correction and right hemispheres are presented for illustration.*

**

***Supplementary Figure S5****. In the placebo condition there were both positive and negative associations between caudate GU and cerebral BOLD during inhibition, while in the secretin condition there was only negative association. Data were thresholded at p<0.05 with FDR cluster-level correction. Cau = caudate, SMA = supplementary motor area, ACC = anterior cingulate cortex, Ins = Insula, Tha = thalamus.*

**

***Supplementary Figure S6****. In the placebo condition there were both positive and negative associations between caudate GU and cerebral BOLD signals in reward response, while in the secretin condition, there was only a positive association. Data were thresholded at p<0.05 with FDR cluster-level correction. Cau = caudate, PCC = posterior cingulate cortex, Ins = Insula.*

**References**

1. Laurila S, Sun L, Lahesmaa M, Schnabl K, Laitinen K, Klén R, et al. Secretin activates brown fat and induces satiation. Nat Metab. 2021;3:798–809.

2. Nummenmaa L, Hirvonen J, Hannukainen JC, Immonen H, Lindroos MM, Salminen P, et al. Dorsal striatum and its limbic connectivity mediate abnormal anticipatory reward processing in obesity. PLoS One. 2012;7.
